# Differential expression of CXCR3 and CCR6 on CD4^+^ T-lymphocytes with distinct memory phenotypes characterizes tuberculosis-associated immune reconstitution inflammatory syndrome

**DOI:** 10.1038/s41598-018-37846-3

**Published:** 2019-02-06

**Authors:** Paulo S. Silveira-Mattos, Gopalan Narendran, Kevan Akrami, Kiyoshi F. Fukutani, Selvaraj Anbalagan, Kaustuv Nayak, Sudha Subramanyam, Rajasekaran Subramani, Caian L. Vinhaes, Deivide Oliveira-de Souza, Lis R. Antonelli, Kumar Satagopan, Brian O. Porter, Alan Sher, Soumya Swaminathan, Irini Sereti, Bruno B. Andrade

**Affiliations:** 1 Instituto Gonçalo Moniz, Salvador, Bahia Brazil; 2 Multinational Organization Network Sponsoring Translational and Epidemiological Research (MONSTER) Initiative, Fundação José Silveira, Salvador, Bahia Brazil; 3 National Institute for Research in Tuberculosis, Chennai, India; 4 Division of Infectious Diseases, Department of Medicine, University of California, San Diego, United States of America; 5 Laboratório de Biologia e Imunologia de Doenças Infecciosas e Parasitárias, Instituto René Rachou, Fundação Oswaldo Cruz, Belo Horizonte, Minas Gerais, Brazil; 6 Government Hospital of Thoracic Medicine, Tambaram, Chennai, India; 7 Clinical HIV Pathogenesis Section, Laboratory of Immunoregulation, National Institute of Allergy and Infectious Diseases, National Institutes of Health, Bethesda, Maryland United States of America; 8 Immunobiology Section, Laboratory of Parasitic Diseases, National Institute of Allergy and Infectious Diseases, National Institutes of Health, Bethesda, Maryland United States of America; 9 Wellcome Trust Centre for Infectious Disease Research in Africa, Institute of Infectious Disease and Molecular Medicine, University of Cape Town, Cape Town, Republic of South Africa; 10 Escola Bahiana de Medicina e Saúde Pública (EBMSP), Salvador, Bahia Brazil; 11 Universidade Salvador (UNIFACS), Laureate Universities, Salvador, Bahia Brazil; 12 Division of Infectious Diseases, Department of Medicine, Vanderbilt University School of Medicine, Nashville, Tennessee United States of America

**Keywords:** T-helper 1 cells, HIV infections, Tuberculosis

## Abstract

Immune reconstitution inflammatory syndrome (IRIS) occurs in up to 40% of individuals co-infected with pulmonary tuberculosis (PTB) and HIV, primarily upon antiretroviral therapy (ART) initiation. Phenotypic changes in T-cells during TB-IRIS and their relationship with systemic inflammation are not fully understood. In this prospective cohort study, we followed 48 HIV-positive patients with PTB from South India before and after ART initiation, examining T-lymphocyte subsets and inflammatory biomarkers in peripheral blood. Quantification of naïve (CD27^+^CD45RO^−^) as well as effector memory CD4^+^ T cells (CD27^−^CD45RO^+^) at weeks 2–6 after ART initiation could distinguish TB-IRIS from non-IRIS individuals. Additional analyses revealed that ART reconstituted different quantities of CD4^+^ T lymphocyte subsets with preferential expansion of CXCR3^+^ CCR6^−^ cells in TB-IRIS patients. Moreover, there was an expansion and functional restoration of central memory (CD27^+^CD45RO^+^) CXCR3^+^CCR6^−^ CD4^+^ lymphocytes and corresponding cytokines, with reduction in CXCR3^−^CCR6^+^ cells after ART initiation only in those who developed TB-IRIS. Together, these observations trace a detailed picture of CD4^+^ T cell subsets tightly associated with IRIS, which may serve as targets for prophylactic and/or therapeutic interventions in the future.

## Introduction

Immune reconstitution inflammatory syndrome (IRIS) is the paradoxical clinical or radiological worsening of a disease or condition that occurs after the initiation of antiretroviral therapy (ART) in HIV infected individuals despite effective virological suppression^1^. IRIS has a propensity to occur when HIV patients are concomitantly infected with other opportunistic pathogens such as *Mycobacterium tuberculosis* (Mtb)^1^. In this setting, previous studies have shown that immune reconstitution triggers aberrant activation of inflammatory responses leading to IRIS^2^. The reported incidence of tuberculosis (TB)-associated IRIS (TB-IRIS) ranges from 2%^3^ to 54%^4^, depending on factors such as the TB endemicity in the region, the degree of immunodeficiency and the mycobacterial antigen load prior to ART initiation^5^.

The pathogenesis of IRIS remains unclear but appears to require two elements: (i) failure of the immune system to eliminate the pathogen (s), leading to persistent and high burden of infection concurrent with (ii) and abrupt immune recovery in response to ART^6^. IRIS is characterized by a heightened and dysregulated activation of pathogen-specific T-lymphocytes. Recent studies, including ours, have shown that frequency of Mtb-specific circulating CD4^+^ T cells against Mtb is intimately associated with onset and occurrence of IRIS^7^ when compared to individuals who do not develop such outcome^8–12^.

Several risk factors have been associated with the development of IRIS such as increased levels of pro-inflammatory cytokines in peripheral blood, as well as degree of lymphopenia prior to ART, the latter being poorly understood^1,13,14^. It is known that lymphocyte depletion alone in the context of HIV and TB occurs due to a direct negative impact on bone marrow as well as apoptosis and lysis of cytotoxic T cells mediated by antibodies^15^.

The detailed participation of T cells in TB-IRIS is not completely described. Since IRIS can sometimes occur prior to quantitative CD4 recovery, functional restoration, rather than a mere increase in T cell number, may play a role in its pathogenesis^8,9,15,16^. Here, we describe TB-IRIS in a TB and HIV treatment naïve population focusing on the relative frequency of various memory and T-helper subsets of CD4^+^ lymphocytes as defined by chemokine receptor expression.

## Results

### TB-IRIS is associated with altered frequencies of naïve and effector memory CD4^+^ T cells

**Figure 1:**
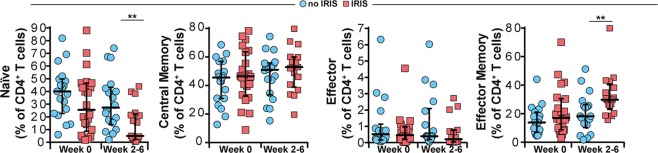
Surface expression of memory markers CD27 and CD45RO on CD4^+^ T-lymphocytes from TB-HIV co-infected patients prior to and following ART initiation. Frequencies of CD27^+^CD45RO^−^ (naïve), CD27^+^CD45RO^+^ (central memory), CD27^−^ CD45RO^−^ (effector) and CD27^−^CD45RO^+^ (effector memory) CD4^+^ T cells from whole blood obtained at week 0 (pre-ART) and at week 6 after ART initiation (in non-IRIS patients, n = 22) or at the time of TB-IRIS event (n = 26). Lines represent median values and interquartile ranges. Data were analyzed using the Mann-Whitney test or Wilcoxon matched-pairs test for paired analyses within each study group. **P < 0.01 after adjustment for multiple measurements.

Surface expression of CD27 and CD45RO was used to define naïve, memory and effector phenotypes in CD4^+^ T cells^7^ in our study population prior to ART initiation and then at 2–6 weeks following treatment. At enrollment pre-ART, the frequency of naïve CD4^+^ T cells (CD27^+^CD45RO^−^) was similar between TB-HIV co-infected patients who developed IRIS and those who did not (Fig. 1). Interestingly, the frequency of these cells was substantially lower in TB-IRIS patients at the time of the IRIS event, compared to non-IRIS patients at equivalent timepoints (Fig. 1). In addition, percentages of both central memory (CD27^+^CD45RO^+^) and effector (CD27^−^CD45RO^−^) cells were not different between TB-IRIS and non-IRIS patients at pre-ART as well as at week 2–6 post-ART initiation (Fig. 1). Of note, the frequency of effector memory CD4 cells (CD27^−^CD45RO^+^) was similar between the study groups at pre-ART but substantially increased during the IRIS events compared to that in non-IRIS patients points after ART initiation (Fig. 1). Our findings indicate that use of CD27 and CD45RO markers on CD4^+^ T cells at pre-ART does not accurately predict and differentiate patients who will develop TB-IRIS from those who will not before ART commencement. However, quantification of naïve and effector memory CD4^+^ T cells after ART initiation could potentially identify TB-IRIS from non-IRIS individuals.

### Antiretroviral therapy initiation leads to substantial alterations of CXCR3 and CCR6 expressing CD4^+^ T cell subsets in TB-HIV co-infected patients

**Figure 2:**
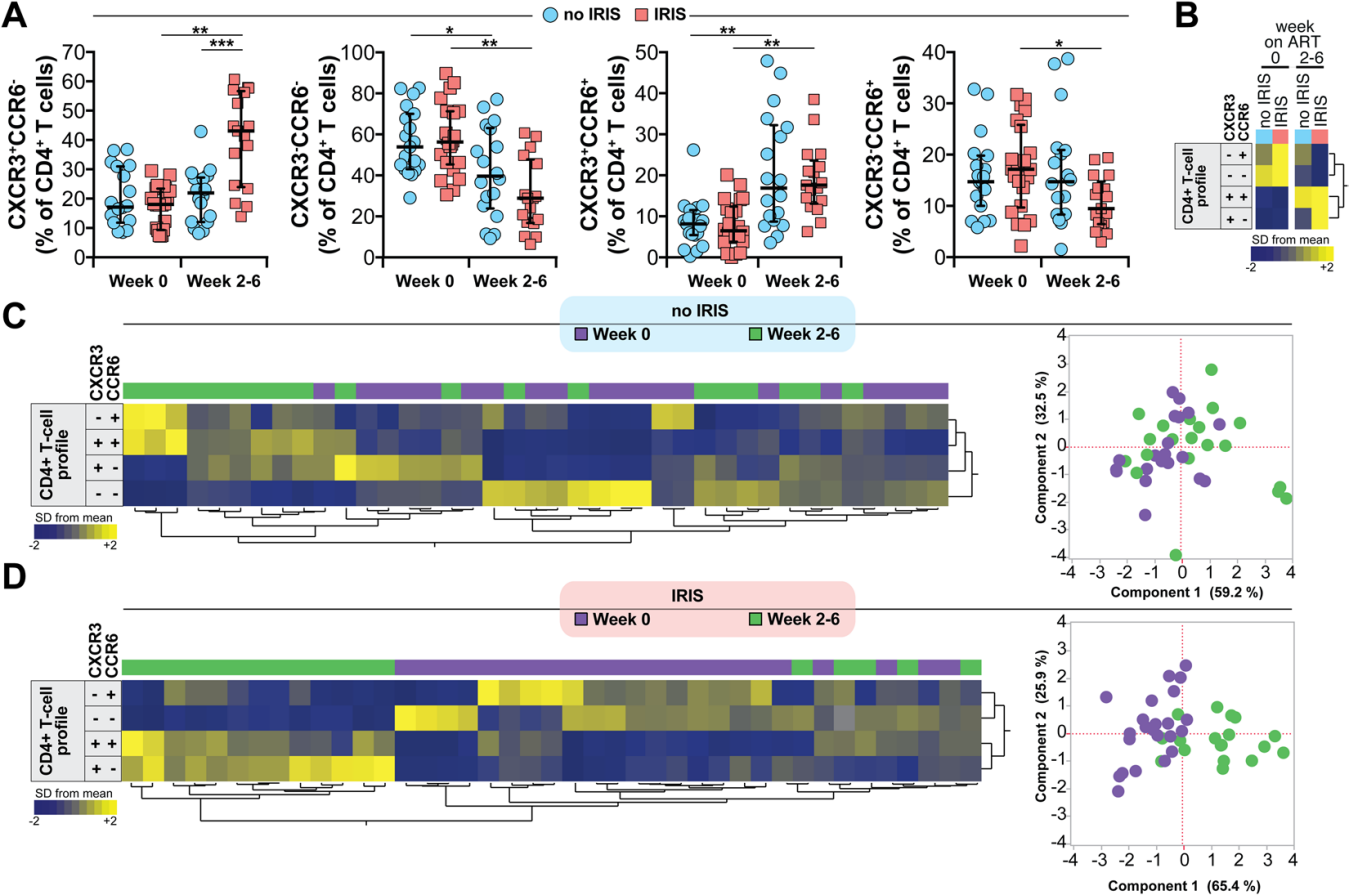
Expression of chemokine receptors CXCR3 and CCR6 in CD4^+^ T-lymphocytes from TB-HIV co-infected patients prior to and following ART initiation. (**A**) Frequency of CXCR3^+^CCR6^−^, CXCR3^−^CCR6^−^, CXCR3^+^CCR6^+^ and CXCR3^−^CCR6^+^ CD4^+^ T cells were evaluated in whole blood obtained at week 0 (pre-ART) and at week 6 after ART initiation (in non-IRIS patients, n = 22) or at the time of TB-IRIS event (n = 26). Lines represent median values and interquartile ranges. Data were analyzed using the Mann-Whitney test or Wilcoxon matched-pairs test for paired analyses within each study group. *P < 0.05, **P < 0.01, ***P < 0.001, after adjustment for multiple measurements. (**B**) Hierarchical cluster analysis of the z-score normalized average frequency values of the CD4^+^ T cell phenotypes is shown to summarize the overall trends of data variation between the study groups and timepoints. Yellow color represents the highest values whereas blue color indicates the lowest values observed for each cell type. Additional hierarchical cluster analyses of the z-score normalized frequency values of the CD4^+^ T cell phenotypes per study participant at pre-ART (**C**) or at week 6 post-ART initiation/time of IRIS event (**D**) were performed to evaluate whether the overall profile of T cell frequencies could differentiate IRIS from non-IRIS patients. These results were confirmed by principal component analysis (PCA) (C and D, left panels).

We next evaluated the differential expression of CXCR3 and CCR6 on T cells. We found that there were major changes in the frequencies of CD4^+^ T cell subsets differentially expressing CXCR3 and CCR6 following ART initiation in this study population (Fig. 2A). In patients who did not develop IRIS, we observed a substantial increase in frequency of CXCR3^+^CCR6^+^ and a significant decrease in the percentage of CXCR3^−^CCR6^−^ the lymphocytes from pre-ART to week 2–6 post-ART initiation (Fig. 2A). In contrast, ART did not impact the frequencies of CXCR3^+^CCR6^−^ and CXCR3^−^CCR6^+^ cells in non-IRIS patients (Fig. 2A). In the group of patients who experienced IRIS during follow-up, we noticed a dramatic increase in the frequencies of both CXCR3^+^CCR6^−^ cells and CXCR3^+^CCR6^+^ lymphocytes (Fig. 2A), while the percentages of CXCR3^−^CCR6^−^ and CXCR3^−^CCR6^+^ cells were significantly diminished (Fig. 2A) upon ART initiation.

We next performed a hierarchical cluster analysis of the median frequencies of CD4 T cells expressing CXCR3 and CCR6 in each study group and time point. This approach revealed that ART initiation was associated with a distinct expression profile independent of the patient groups (Fig. 2B). Further analyses using hierarchical clustering of individual values, as well as a principal component analysis (PCA), indicated that in non-IRIS patients, the differential expression of CXCR3 and CCR6 could not distinguish the study timepoints (pre-ART and week 2–6 post-ART initiation) (Fig. 2B). In the group of TB-IRIS patients, the frequencies of the different CD4^+^ T cell subsets examined were distinct between the study time points (Fig. 2C). These findings argue that while the frequencies of CD4^+^ T cell subsets differentially expressing CXCR3 and CCR6 are affected by ART, there is a unique expansion of CXCR3^+^CCR6^−^ and CXCR3^+^CCR6^+^ cells in TB-IRIS patients after ART initiation.

### Assessment of CXCR3, CCR6 expression and memory cell markers identifies IRIS events after ART initiation

**Figure 3:**
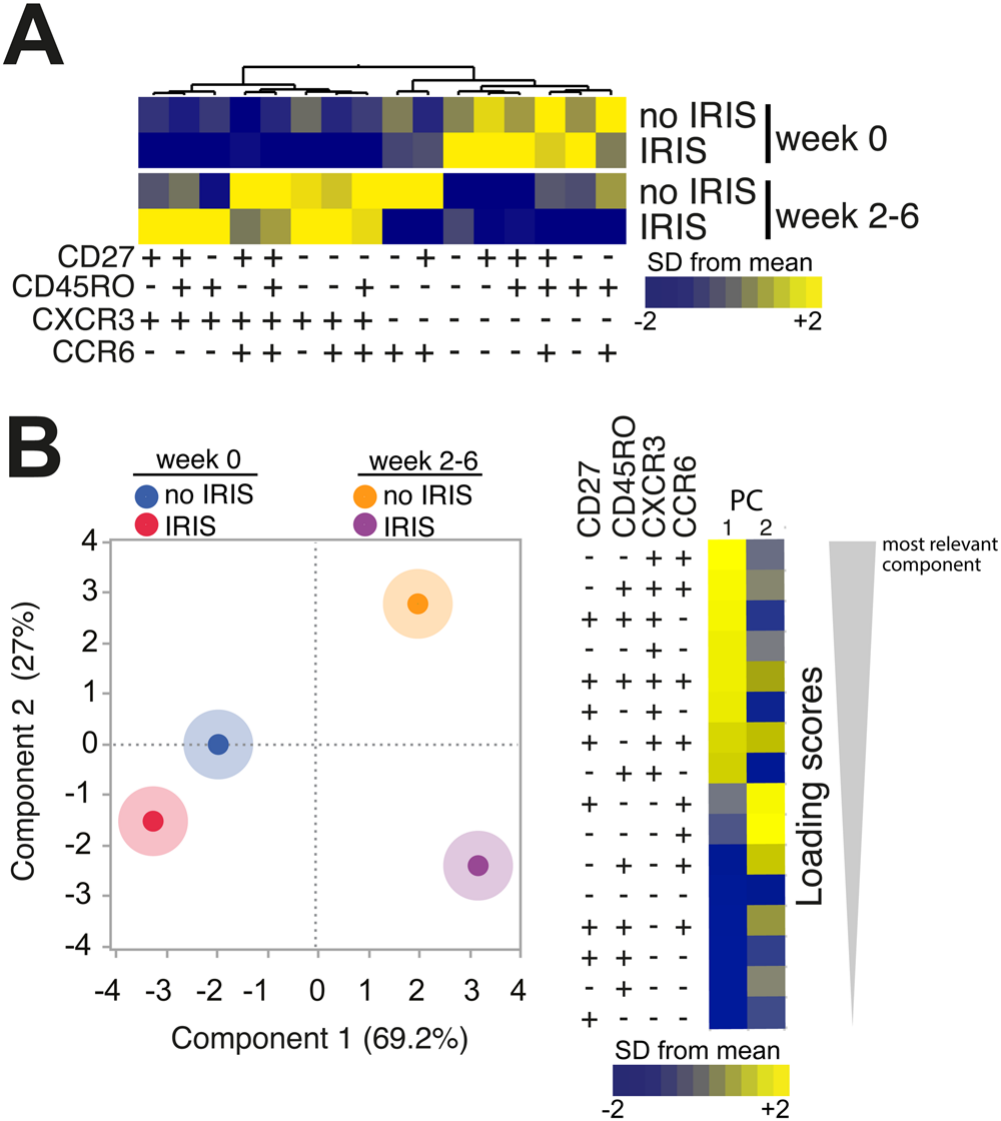
TB-IRIS patients can be distinguished from non-IRIS based on simultaneous assessment of memory markers and chemokine receptors. (**A**) Hierarchical cluster analysis of the z-score normalized average frequency values of the indicated CD4^+^ T cell phenotypes is shown to summarize the overall trends of data variation between the study groups and timepoints. Yellow color represents the highest values whereas blue color indicates the lowest values observed for each cell type. (**B**) A PCA model was employed to test whether a combination of the memory cell markers and chemokine receptors could cluster patients with IRIS vs. non-IRIS at week 0 (pre-ART) and at week 6 post-ART initiation (right panel). Loading Scores reflect the strength that the combination of markers represents in each principal component shown (left panel).

We tested the combination of memory/naïve and chemokine receptor markers that could better characterize TB-IRIS. A hierarchical cluster analysis of the median frequencies of cells expressing CD27, CD45RO, CXCR3 and CCR6 indicated that ART initiation selectively led to changes in the expression profiles in both groups of IRIS and non-IRIS patients (Fig. 3A). Furthermore, a PCA model indicated that the overall expression profile of these cell surface markers was similar between IRIS and non-IRIS groups at pre-ART, whereas important differences were evident at week 2–6 post ART between the IRIS and non-IRIS groups (Fig. 3B). The most significant variables driving these differences were the frequencies of effector and effector memory CXCR3^+^CCR6^+^cells as well as central memory CXCR3^+^CCR6^−^ lymphocytes (Fig. 3B). This finding suggests that these cells were highly associated with the occurrence of TB-IRIS in TB-HIV co-infected patients initiating ART.

### Expansion of CXCR3^+^CCR6^−^ and of central memory CXCR3^−^CCR6^+^ lymphocytes is strongly associated with systemic inflammation typical of IRIS in TB-HIV co-infected patients receiving ART

**Figure 4:**
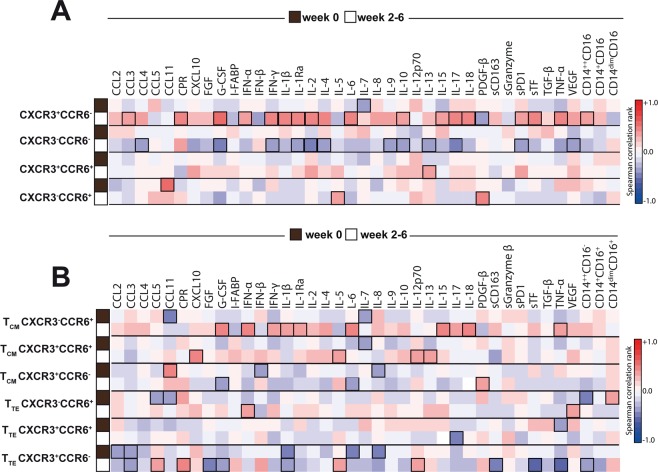
Dynamics of associations between CD4^+^ T cell subtypes with circulating levels of several pro-inflammatory cytokines and chemokines, as well as frequency of monocyte subtypes. (**A**) Frequencies of CXCR3^+^CCR6^−^, CXCR3^−^CCR6^−^, CXCR3^+^ CCR6^+^ and CXCR3^−^CCR6^+^ lymphocytes were tested for correlations with several pro-inflammatory cytokines and chemokines at week 0 (pre-ART) and at week 6 post-ART initiation or at the time of IRIS event in the entire study population (n = 48). (**B**) Frequencies of CXCR3^+^CCR6^−^, CXCR3^+^ CCR6^+^ and CXCR3^−^CCR6^+^ CD4^+^ T-lymphocytes expressing markers of central memory (CM) or T effector (TE) cells were tested for correlations with the same parameters shown in (**A**). A heat map was used to represent the strength of the associations (Spearman rank value). Statistically significant correlations (P < 0.05) after adjustment for multiple measurements are highlighted with bold squares.

We next ascertained whether subsets of CD4^+^ T cells described above are correlated with plasma levels of a large panel of cytokines, chemokines and growth factors, frequencies of monocyte subsets, in the entire study population (n = 48) before and after ART initiation. Prior to ART initiation, there were no significant correlations between the frequency of CD4^+^ T cell subsets and monocyte frequencies or with plasma biomarker concentrations (Fig. 4A,B), except for interleukin (IL)-7, which was negatively correlated with frequency of CXCR3^+^CCR6^−^ cells. In contrast, frequency of CXCR3^+^CCR6^−^ cells at week 2–6 of ART exhibited positive correlations with frequency of pro-inflammatory monocytes (CD14^++^CD16^−^) as well as levels of several key mediators of inflammation, including C-reactive protein (CRP), IL-6, interferon (IFN)-γ, IL-1β, IL-17, IL-18, soluble tissue factor (sTF) and tumor necrosis factor (TNF)-α (Fig. 4A). In addition, the percentage of central memory CXCR3^+^CCR6^−^ cells was also directly associated with systemic concentrations of pro-inflammatory mediators, including type I and II IFNs, IL-1β, IL-1Ra, IL-6, IL-15, IL-18 and TNF-α (Fig. 4B).

**Figure 5:**
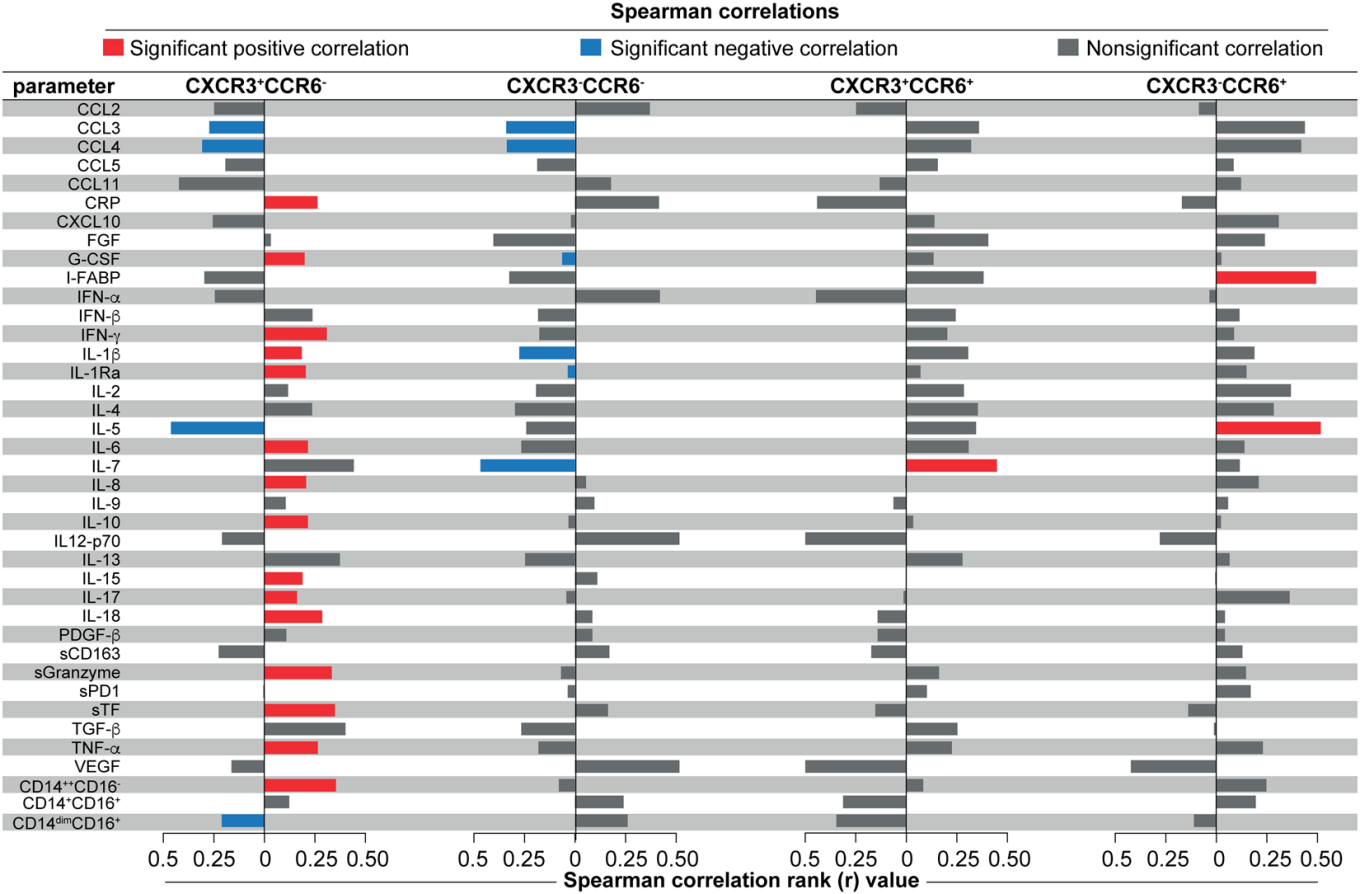
Associations between changes in systemic inflammation between before and after ART commencement and frequencies of CD4^+^ T cell subtypes. For the entire study population, change in inflammation over time was estimated by calculating fold-differences of concentrations of each indicated plasma markers as well as for frequencies of the monocyte subsets, from pre-ART to week 2–6 after ART initiation (values at week 2–6 divided by values from pre-ART). Spearman analysis was used to test correlations between changes values of the inflammatory markers and frequencies of the indicated CD4+ T cell subsets in peripheral blood at the time of TB-IRIS event or equivalent timepoint. Colored bars indicate statistically significant correlations (p < 0.05) after adjustment for multiple measurements.

More detailed analyses were performed to better understand the relationships between changes in concentrations of inflammatory markers and frequencies of monocyte subsets from pre-ART to time of TB-IRIS event or equivalent timepoint and frequencies of the distinct CD4^+^ T cell subsets depicted above. Using Spearman correlation matrices, we found that increases in levels of innate and adaptive immune activation markers were associated with frequencies of CXCR3^+^CCR6^−^ cells, but not of the other subsets (Fig. 5). Interestingly, changes in monocytes subsets, previously described as predictors of TB-IRIS^17^, were associated with increased frequencies of CXCR3^+^CCR6^−^ cells in peripheral blood after ART initiation (Fig. 5). Indeed, increased frequency of CD14^++^CD16^−^ pro-inflammatory monocytes from baseline to week 2–6 of ART positively correlated with the proportion of CXCR3^+^CCR6^−^ cells in blood during the latter time point. On the converse, increasing frequency of the patrolling monocytes (CD14^dim^CD16^+^) during the first weeks after ART commencement was negatively associated with proportion of CXCR3^+^CCR6^−^ cells at the onset of IRIS event or equivalent time point (Fig. 5). These findings strongly suggest that CXCR3^+^CCR6^−^ CD4^+^ T cells participate in the intricate pro-inflammatory changes, which occur upon immune reconstitution and favor occurrence of TB-IRIS.

## Discussion

Our study highlighted the concept that differential expression of CXCR3 and CCR6 on effector and memory CD4^+^ T cells was associated with development of TB-IRIS in HIV patients following ART initiation and can be linked with the inflammatory milieu, both soluble mediators and inflammatory monocytes, that characterizes this syndrome.

Phenotypic analysis of circulating CD4^+^ T cells showed a higher frequency of effector memory (CD27^−^ CD45RO^+^) T cells and a decreased frequency of naïve (CD27^+^ CD45RO^−^) T cells in IRIS compared to non-IRIS patients at equivalent time points after ART initiation. While this difference could reflect advanced HIV infection, which is an important risk factor for IRIS subsequently developing in the presence of antigenemia as in TB^18^, differences among CD4^+^ T cell types were negligible at pre-ART in both groups. Alterations in the frequency of circulating memory T cells have been reported in TB-IRIS^19–22^. Haridas *et al*. demonstrated that the post-ART/TB-IRIS shift of the CD4^+^ T cell memory compartment to an effector memory-dominated phenotype could help in controlling acute TB infection during the early stages of ART-mediated immune restoration, thereby conferring long-term enhanced protection against Mtb reinfection/reactivation/relapse^20^.

Our findings complement those of Antonelli *et al*. studying a US patient cohort who described a higher proportion of effector cells at the time of an IRIS event and at 6 months post-ART and a higher percentage of naïve cells in the non-IRIS group. This reiterates the fact that non-IRIS patients reconstitute the naïve cell compartment faster while IRIS patients expanded initially predominantly CD4^+^ effector T cells^7^. This is not unexpected considering that persistent antigenemia in TB could facilitate the expansion and survival of effector cells which are antigen-specific. Consistent with our observations, chronic and indolent infection (such as TB) with persistent antigenemia can provide the ideal environment and stimulus for the persistence of effector T cells.

It is well established that the differential expression profile of CXCR3 and CCR6 can define distinct T helper (Th) phenotypes^23,24^. Following this concept, Th1 cells are usually defined as population with CXCR3^+^CCR6^−^, whereas CXCR3^−^CCR6^−^ are hallmarks of Th2 cells. Recently, a new, CXCR3^+^CCR6^+^ CD4^+^ subset referred to as Th1* has been described that appears to play a critical role in mycobacterial infections in humans^25^. Lastly, Th17 lymphocytes, defined as CXCR3^−^CCR6^+^, have also been implicated in TB immune responses and pathogenesis^26^. Although we have not directly tested cytokine production, the chemokine expression analyses reinforce the idea that a predominantly Th1 type of response and associated cytokine outburst underlies the clinical presentation of IRIS with a corresponding decline in Th2 response, more pronounced among IRIS patients compared to non-IRIS patients after ART initiation. Furthermore, we noticed a dramatic increase in the frequencies of Th1 and Th1* lymphocytes, while the percentages of Th2 and Th17 cells were significantly diminished upon ART initiation^27^. Th phenotypic characterization was equivalent in both the IRIS and non-IRIS groups at baseline prior to ART initiation, thereby aiding in the diagnosis of IRIS but not in its prediction.

Our study reaffirms that the tilt in balance from a Th2 to Th1 immune phenotype with ART administration may occur in IRIS. We previously demonstrated that after ART initiation, patients who experienced IRIS exhibit hyperactivation of T cells specific to antigens from opportunistic pathogens, leading to elevated levels of many pro- and anti-inflammatory cytokines and chemokines, resulting in a phenomenon known as cytokine storm or hypercytokinemia^28^. Consistent with our study, Meintjes *et al*. showed a higher frequency of IFN-γ secreting Th1 cells in patients with IRIS compared to non-IRIS patients, which induces the cytokine storm^29^.

Prior studies have also demonstrated that in IRIS, there is an increase in Th1 cytokines such as IL-2, IL-12, IFN-γ and TNF-α^8,30^. Acute exacerbation of Mtb-specific Th1 responses were independent of CD4^+^ T cell count, viral load and time of ART initiation^8^. This supports the idea that, rather than raising T cell numbers, ART contributes to a functional restoration of these cells. Quantification of CD4^+^ T cells may not reflect the true cell count, as there may be transient sequestration of inflammatory cells at the tissue level that was not detected or a delayed increase in the frequency of these cells^15^.

The role of innate immunity in the pathogenesis of TB-IRIS has been studied by several groups worldwide. There is strong indication that innate immune activation prior to ART commencement, with elevated levels of IL-6^4^ and IL-18^31^ followed by inflammasome activation^32–34^ as well as expansion of inflammatory monocyte subsets^17^, hallmarks patients at higher risk of IRIS. Whether pre-ART dysregulation of innate immune activation contributes to abnormal T-cell activation during IRIS is unknown. In the present study, we found that between the timepoints examined (baseline and week 2–6 after ART commencement), increases in plasma levels of inflammatory markers such as CRP, G-CSF, IL-1β, IL-1Ra, IL-6, IL-8, IL-18, TNF-α and sTF were directly associated with frequencies of CXCR3^+^CCR6^−^ CD4^+^ T cells in the study population. This scenario was also associated with increases in proportion of CD14^++^CD16^−^ monocytes in peripheral blood. These observations make possible to hypothesize that expansion of CXCR3^+^CCR6^−^ CD4^+^ T cells, rather than representing the main immunological basis of TB-IRIS, may be driven by an underlying augmentation of pro-inflammatory innate mediators prior to ART in TB-HIV patients with high microbial burden and who are at a very high risk of developing this syndrome.

The strengths of the current study were the selection of a uniform group of patients who had culture-positive, drug sensitive pulmonary TB, were naïve at baseline for anti-tuberculous treatment and ART, had an increase in their CD4^+^ T cell population with immune recovery, and demonstrated a decline in HIV viral concentration of at least 0.5 log after the initiation of ART. With complete mycobacterial culture and drug sensitivity results, we excluded the possibility of TB treatment failure at the time of IRIS diagnosis by demonstrating negative cultures at IRIS diagnosis, thereby strengthening the validity of our findings. Since all patients were admitted during ART initiation, there was active surveillance for IRIS with clinical samples collected immediately after the onset of IRIS prior to the institution of anti-inflammatory agents, thereby avoiding distortion of lab parameters or T cell subsets. The main limitation of our study was the relatively small number of patients analyzed as the cohort was nested within a randomized controlled trial with stringent inclusion and exclusion criteria that precluded the recruitment and analysis of a larger sample.

Finally, in the current study we evaluated the expression of chemokine receptors and memory markers in CD4^+^ T cells, along with their association with plasma biomarkers and monocyte subtypes that were shown to accurately help to diagnose IRIS. These findings confirm a prominent role of ART and Th1 effector cells in pathogenesis of IRIS.

## Methods

### Description of the patients

All clinical investigations were conducted according to the principles expressed in the Declaration of Helsinki. Written informed consent was obtained from all study participants. This study was approved by the Scientific Advisory Committee and Institutional Ethics Committee of the National Institute for Research in Tuberculosis (Chennai, India) and the main randomized clinical trial that provided patient information and samples for the present study was registered on Clinicaltrials.gov (NCT00933790).

### Description of the patients

The Indian TB-IRIS cohort study was a retrospective observational analysis of cryopreserved samples from an investigation nested within a randomized controlled trial (NCT00933790) at the National Institute for Research in Tuberculosis (NIRT), Chennai, India, enrolling HIV-infected patients with newly diagnosed sputum culture-confirmed pulmonary TB, as previously reported. The parent randomized controlled clinical trial compared daily vs. intermittent anti-TB therapeutic regimens in HIV infected patients with pulmonary TB and has been already published^35^. Eligible participants in the TB-IRIS observational study were above 18 years of age, with newly diagnosed culture-positive rifampicin-sensitive TB, and who were ART-naïve^4^. They were initiated on ART within the intensive phase of anti-tuberculous therapy, as per prevailing national guidelines (National AIDS Control Organization, NACO). Clinical evaluations and blood collections were performed at baseline (pre-ART), at the time of the IRIS event (between weeks 2–6 post-ART initiation) or after 6 weeks of ART in the non-IRIS group, and after 6 months of ART in both groups. Mycobacterial loads in sputum cultures were assessed as described elsewhere^4^. IRIS was diagnosed by a panel of 3 doctors after ruling out drug resistance, as well as failure and occurrence of other opportunistic infections or common endemic infections. Modified INSHI criteria that included a 0.5 log decline in HIV viral load from baseline at the time of IRIS and a negative mycobacterial culture or decline in grade of TB infection from baseline were added for a definitive diagnosis of IRIS^18^. All patients were hospitalized for ART initiation and were discharged within two weeks. In this cohort, 48 individuals were enrolled and 26 (54%) developed IRIS during the study. The detailed clinical, laboratory, and microbiologic description of the study participants has been previously reported by our group^4^.

### Plasma biomarker measurements

Concentrations of IL-1β, IL-1Ra, IL-6, IL-8, IL-10, IL-12p40, IL-12p70, IL-15, IL-17, IL-18, CCL2, CCL3, CCL4, CCL5, CCL11, CXCL10, IFN-γ, TNF-α, TGF-β, platelet-derived growth factor (PDGF), vascular endothelial growth factor (VEGF) (Bio-Plex, Bio-Rad, Hercules, CA), C-reactive protein (CRP) (eBioscience, San Diego, CA), sCD163, soluble tissue factor (sTF) (R&D Systems, Minneapolis, MN) and intestinal fatty acid binding protein (I-FABP) (Hycult Biotech, The Netherlands) were assessed in cryopreserved plasma samples maintained at −80 °C.

### Flow cytometry

The immunophenotyping of lymphocytes and monocytes was performed in whole blood collected in heparinized vacuum tubes. For “*ex vivo*” phenotyping, aliquots of 250 µL blood were stained with five panels of antibodies prepared in PBS 1% BSA for one hour at room temperature (RT) to characterize the lymphocyte populations. The panels with antibody clones and fluorochromes, as well as the gating strategies, are listed in Fig. S1. Antibodies were from eBioscience (San Diego, CA), Biolegend (San Diego, CA), BD Biosciences (San Jose, CA) and Life Technologies (Carlsbad, CA). Data were acquired on a BD FACS Canto II flow cytometer (BD Biosciences). The panel of T cells was defined based on surface expression of CD3 and CD4, different memory subpopulations to discriminate naïve, central and effector memory T cells using CD27 and CD45RO. The chemokine receptor expression was characterized used CXCR3 and CCR6. The immunophenotyping of subsets of monocytes was described previously^17^. All compensation and gating analyses were performed using FlowJo 9.5.3 (TreeStar, Ashland, OR).

### Data analysis

Median values with IQR or frequencies of variables were compared using the Mann-Whitney test (when two groups were compared) or the Kruskal-Wallis test with Dunn’s multiple comparisons ad hoc analysis (when three groups were compared). Fisher’s exact test or Chi-square tests were used to compare two or three groups, respectively, for proportions. Paired changes from before ART initiation to week 6 or the time of IRIS development were compared using the Wilcoxon matched-paired T test. Using JMP 10.0 software, geometric mean values (log10) for each marker measured at week 0 and week 6 were calculated for the entire study population. To assess the overall pattern of expression of these markers in each clinical group and time point, heat maps were built using variation from the geometric mean value calculated for each candidate biomarker. A hierarchical cluster analysis using the Ward’s method was employed to reveal patterns of expression in plasma. Throughout the text, a p value of <0.05 was taken as statistically significant after adjustments for multiple measurements (Holm-Bonferroni’s correction method). The statistical analyses were performed using GraphPad Prism 6.0 (GraphPad Software Inc., USA), STATA 9.0 (StataCorp, TX, USA), and JMP 10.0 software.

## Supplementary information

Supplementary Figure 1
