# Author Correction: Differential expression of CXCR3 and CCR6 on CD4^+^ T-lymphocytes with distinct memory phenotypes characterizes tuberculosis-associated immune reconstitution inflammatory syndrome

**DOI:** 10.1038/s41598-019-44429-3

**Published:** 2019-05-24

**Authors:** Paulo S. Silveira-Mattos, Gopalan Narendran, Kevan Akrami, Kiyoshi F. Fukutani, Selvaraj Anbalagan, Kaustuv Nayak, Sudha Subramanyam, Rajasekaran Subramani, Caian L. Vinhaes, Deivide Oliveira-de Souza, Lis R. Antonelli, Kumar Satagopan, Brian O. Porter, Alan Sher, Soumya Swaminathan, Irini Sereti, Bruno B. Andrade

**Affiliations:** 1 Instituto Gonçalo Moniz, Salvador, Bahia Brazil; 2 Multinational Organization Network Sponsoring Translational and Epidemiological Research (MONSTER) Initiative, Fundação José Silveira, Salvador, Bahia Brazil; 3 National Institute for Research in Tuberculosis, Chennai, India; 4 Division of Infectious Diseases, Department of Medicine, University of California, San Diego, United States of America; 5 Laboratório de Biologia e Imunologia de Doenças Infecciosas e Parasitárias, Instituto René Rachou, Fundação Oswaldo Cruz, Belo Horizonte, Minas Gerais Brazil; 6 Government Hospital of Thoracic Medicine, Tambaram, Chennai India; 7 Clinical HIV Pathogenesis Section, Laboratory of Immunoregulation, National Institute of Allergy and Infectious Diseases, National Institutes of Health, Bethesda, Maryland United States of America; 8 Immunobiology Section, Laboratory of Parasitic Diseases, National Institute of Allergy and Infectious Diseases, National Institutes of Health, Bethesda, Maryland United States of America; 9 Wellcome Trust Centre for Infectious Disease Research in Africa, Institute of Infectious Disease and Molecular Medicine, University of Cape Town, Cape Town, Republic of South Africa; 10 Escola Bahiana de Medicina e Saúde Pública (EBMSP), Salvador, Bahia Brazil; 11 Universidade Salvador (UNIFACS), Laureate Universities, Salvador, Bahia Brazil; 12 Division of Infectious Diseases, Department of Medicine, Vanderbilt University School of Medicine, Nashville, Tennessee United States of America

Correction to: *Scientific Reports* 10.1038/s41598-018-37846-3, published online 06 February 2019

In the original version of this Article, Irini Sereti was incorrectly affiliated with ‘Government Hospital of Thoracic Medicine, Tambaram, Chennai, India’. The correct affiliation is listed below.

Clinical HIV Pathogenesis Section, Laboratory of Immunoregulation, National Institute of Allergy and Infectious Diseases, National Institutes of Health, Bethesda, Maryland, United States of America

This error has now been corrected in the PDF and HTML versions of the Article.

