# Supplementary material for: Differential expression of CXCR3 and CCR6 on CD4+ T-lymphocytes with distinct memory phenotypes characterizes tuberculosis-associated immune reconstitution inflammatory syndrome
Source: Sci Rep. 2019 Feb 6;9:1502. doi: 10.1038/s41598-018-37846-3 (PMC6365576; doi:10.1038/s41598-018-37846-3)
Supplement: Supplementary file 1 — Supplementary Figure 1 [file 41598_2018_37846_MOESM1_ESM.pdf]

Differential expression of CXCR3 and CCR6 on CD4$^+$ T-lymphocytes with distinct memory phenotypes characterizes tuberculosis-associated immune reconstitution inflammatory syndrome

Paulo S. Silveira-Mattos, Gopalan Narendran, Kevan Akrami, Kiyoshi F. Fukutani, Selvaraj Anbalagan, Kaustuv Nayak, Sudha Subramanian, Rajasekaran Subramani, Caian L. Vinhaes, Deivide O. Souza, Lis R. Antonelli, Kumar Satagopan, Brian O. Porter, Alan Sher, Soumya Swaminathan, Irini Sereti, Bruno B. Andrade

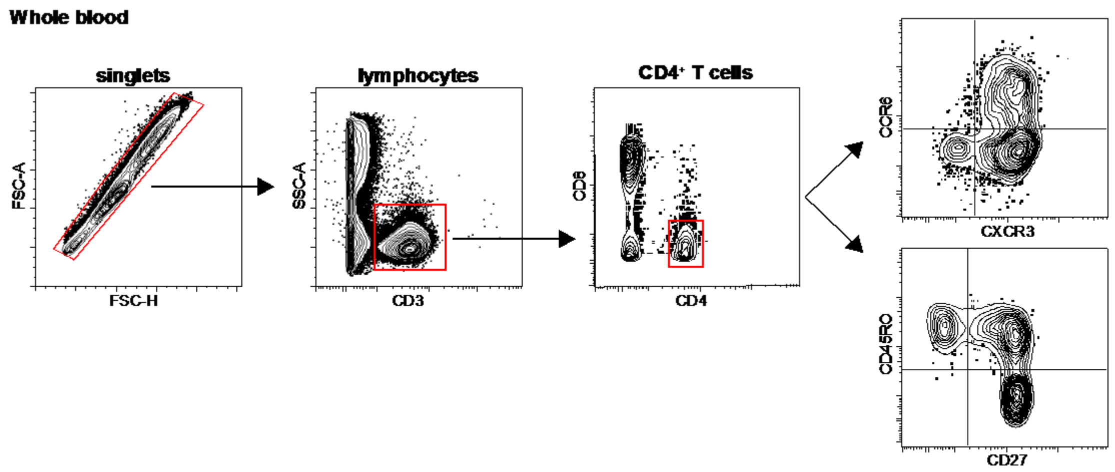

Figure S1. Overall gating strategy.
